# Serum Uric Acid Is Associated with Poor Outcome in Black Africans in the Acute Phase of Stroke

**DOI:** 10.1155/2017/1935136

**Published:** 2017-09-10

**Authors:** Yacouba N. Mapoure, Chia Mark Ayeah, M. S. Doualla, H. Ba, Hugo B. Mbatchou Ngahane, Salomon Mbahe, Henry N. Luma

**Affiliations:** ^1^Department of Clinical Sciences, University of Douala, Douala, Cameroon; ^2^Department of Internal Medicine, Douala General Hospital, Douala, Cameroon; ^3^Department of Internal Medicine, University of Yaoundé I, Douala, Cameroon

## Abstract

**Background:**

Prognostic significance of serum uric acid (SUA) in acute stroke still remains controversial.

**Objectives:**

To determine the prevalence of hyperuricemia and its association with outcome of stroke patients in the Douala General Hospital (DGH).

**Methods:**

This was a hospital based prospective cohort study which included acute stroke patients with baseline SUA levels and 3-month poststroke follow-up data. Associations between high SUA levels and stroke outcomes were analyzed using multiple logistic regression and survival analysis (Cox regression and Kaplan-Meier).

**Results:**

A total of 701 acute stroke patients were included and the prevalence of hyperuricemia was 46.6% with a mean SUA level of 68.625 ± 24 mg/l. Elevated SUA after stroke was associated with death (OR = 2.067; 95% CI: 1.449–2.950;* p* < 0.001) but did not predict this issue. However, an independent association between increasing SUA concentration and mortality was noted in a Cox proportional hazards regression model (adjusted HR = 1.740; 95% CI: 1.305–2.320;* p* < 0.001). Furthermore, hyperuricemia was an independent predictor of poor functional outcome within 3 months after stroke (OR = 2.482; 95% CI: 1.399–4.404;* p* = 0.002).

**Conclusion:**

The prevalence of hyperuricemia in black African stroke patients is quite high and still remains a predictor of poor outcome.

## 1. Introduction

Stroke is the second most common cause of death and major cause of disability worldwide [1]. The burden of stroke is increasing in many low- and middle-income countries (LMIC) [2], and due to the high fatality rates and overwhelming resources incurred by health systems, stroke and many noncommunicable diseases (NCDs) are now targeted public health priorities in these regions [3, 4]. SUA is one of the most important antioxidants abundant in the plasma, contributing to approximately two-thirds of free radical scavenging in plasma [5, 6]. SUA has been concerned as a free radical scavenger protecting nerves from oxidative injury [7, 8]. A study explored the relationship between SUA levels and 1-year outcomes, and vascular events of acute ischemic stroke patients showed that decreased SUA levels were related to poor outcomes and had a higher risk of all-cause death [9]. Meanwhile, Amaro et al. suggested that increased SUA is associated with better outcomes in patients with stroke treated with reperfusion therapies and supported the assessment of the potential neuroprotective role of the exogenous administration of UA in patients with stroke [10]. These findings suggested that SUA may be neuroprotective in acute ischemic stroke patients. Conversely, some studies have explored the relationship between SUA and stroke prognosis but have had contradicting results. Previous experimental studies in recent years have reported a relationship between SUA and several cardiovascular conditions including acute myocardial infarction (AMI), congestive heart failure (CHF), and both ischemic and hemorrhagic stroke [11–14]. These studies suggested that increasing SUA was associated with a higher risk of mortality. Despite previous and current studies, the neuroprotective role of SUA in stroke remains controversial. In this present study, our objectives were to determine the prevalence of hyperuricemia and to examine the association of SUA levels with CVRFs and outcomes of stroke patients in the acute phase.

## 2. Methods and Patients

### 2.1. Patients and Study Design

We carried out a hospital based prospective cohort study in a tertiary care hospital in Douala, Cameroon. We included consenting patients admitted for acute stroke (both ischemic and hemorrhagic) in the Neurology Unit of the Department of Internal Medicine and the Intensive Care Unit of the Douala General Hospital (DGH) from January 2010 to January 2016. This study was approved by the Institutional Ethics Committee of Research on Human Health of the University of Douala and the study hospital DGH. Patients who were admitted for confirmed acute stroke within 7 days of onset of symptoms were included in our study. Patients with cerebral venous thrombosis and subarachnoid hemorrhage were excluded.

### 2.2. Data Collection and Patient Management

Demographic data, including age and sex, and relevant medical history such as hypertension (HTN), diabetes mellitus (DM), smoking history, alcohol abuse, use of diuretics, history of diseases like chronic kidney disease (CKD), and gout and other cardiovascular events such as atrial fibrillation, congestive heart failure (CHF), coronary artery disease (CAD), and ischemic heart disease (IHD) were recorded. Baseline vital and anthropometric parameters such as blood pressure, pulse, respiratory rate, oxygen saturation, weight, height, and abdominal circumference values were recorded using standard operating procedures. Hypertension, diabetes mellitus, dyslipidemia, alcohol consumption, and obesity were defined as in the previous study published in this journal by Mapoure et al. in 2014 [15]. Metabolic syndrome was defined as per the NCEP ATP III guidelines [16]. Patients with severe conditions like a Glasgow Coma Scale <8/15 or septic shock were directly admitted in the ICU while other cases were hospitalized in the NU.

Blood samples were collected from all patients during the first 24 hours of admission to check SUA levels, fasting blood sugar, complete metabolic panel (urea, creatinine, uric acid, and electrolytes), and lipid profile using the Cobas 311 autoanalyzers. A full blood count with platelet counts, prothrombin time, kaolin cephalin clotting time, C-reactive protein (CRP), erythrocyte sedimentation rate (ESR), and HIV serology was done. Other tests were prescribed if required by the patient's conditions: chest X-ray, urine culture, hemoculture, and thick blood film to check for* Plasmodium falciparum*. Neurological assessment was done by a neurologist or intensive care specialist. Interpretation of CT scans was done by both a radiologist and a neurologist and its findings were recorded. The type of stroke and ischemic stroke subtypes was also recorded. Electrocardiography was systematically done for patients with ischemic stroke and for hypertensive patients with hemorrhagic strokes. For patients with ischemic stroke, transthoracic and supra-aortic Doppler ultrasound was done, except for those with severe conditions. On admission and at 3 months after stroke, the Glasgow Coma Scale (GCS) and the National Institute of Health Stroke Scale (NIHSS) were used to determine the stroke severity while the functional outcome was evaluated by the Modified Rankin Score (mRS). Stroke death and stroke recurrence during admission and within 3 months after stroke were also recorded. Poor functional outcome was considered in patients with mRS >2 within the first 3 months after stoke discharge. Follow-up was done daily for clinical evaluation and complications were noted. Oxygen was administrated if ambient oxygen saturation was less than 94%. Paracetamol was used at 1 g every six hours if temperatures were above 37.5°C. Prevention of deep venous thrombosis and stress ulcers was done using a prophylactic dose of enoxaparin (40 mg) and omeprazole (20 mg), respectively. An insulin protocol was set up when capillary glycemia was above 1.4 g/L. Concerning the blood pressure, nicardipine was given intravenously with an electric syringe in case of high blood pressure with a target of 140 to 160 mmHg for systolic blood pressure in hemorrhagic stroke. In ischemic stroke, early elevated blood pressure was respected except when this was more than 220 mmHg for systolic pressure. Aspirin (100–250 mg per day) was given in ischemic stroke while the curative dose of low weight molecular heparin (0.1 mL/kg twice daily) was used in case of atrial fibrillation with CHADS VASC score >2, presence of intraluminal thrombus in a cerebral artery, or presence of blood clot in the heart. Antibiotics and artemether were used for bacterial infection and malaria, respectively. Thrombolysis is not yet effective in Cameroon.

### 2.3. Statistical Analysis

Statistical analysis was done using the Statistical Package for the Social Sciences (SPSS) standard version, Release 20.0 (IBM Inc., 2012). Mean and standard deviation (SD) of all continuous data are reported. SUA was a scale variable but was categorized into two groups, those having normal and high SUA levels (hyperuricemia) as follows: normal SUA range was SUA ≤ 60 mg/l in females and SUA ≤ 70 mg/l in males while high SUA range was SUA > 60 mg/l in females and SUA > 70 mg/l in males. SUA was also divided into quintiles (mean SUA level per quintile range) as follows: *Q*1 ≤ 49 mg/l (41.92 mg/l), *Q*2 = 50–60 mg/l (55.41 mg/l), *Q*3 = 61–70 mg/l (65.83 mg/l), *Q*4 = 71–84 mg/l (76.91 mg/l), and *Q*5 ≥ 85 mg/l (105.45 mg/l). Independent samples *t*-test was used to assess differences in continuous variables since the normality assumption was not violated following Kolmogorov-Smirnov and Shapiro-Wilk test for normality. Cramer's *V*, chi-square test, and Fisher's exact test were used for categorical variables. Univariate analysis was first performed with demographic characteristics and the cerebrovascular risk factors by cross-tabulations with *X*^2^ or Fisher's exact tests for the unadjusted odds ratios (ORs) and then multiple logistic regression was done to adjust the confounding effects of the dependent predictors of death. All predictor variables with *p* values < 0.2 that were purposefully gotten from the univariate analysis were included in our multivariate analysis. Survival analysis was performed using Kaplan-Meier and Cox regression analysis. Level of significance was considered 0.05 (two-sided).

## 3. Results 

### 3.1. Baseline Characteristics of Patients

We recruited a total of 701 acute stroke patients among whom 389 (55.5%) were male patients giving a M : F ratio of 1.25 : 1 (5 : 4). The age range was from 23 to 100 years with a mean age of 60.58 ± 13.27 years. Of the 701 participants, 480 (68.5%) had ischemia. 18 (2.6%) out of 480 ischemic stroke patients presented with a transient ischemic attack (TIA) on admission. HTN was the most predominant CVRF in 664 (94.7%). 523 (74.6%) out of the 664 patients with HTN had a known history of HTN while 141 (20.1%) were not known to be hypertensive patients but had acutely elevated blood pressure >130/85 mmHg and even required antihypertensive agents during admission. HTN was insignificantly more predominant among ischemic stroke patients (450, 93.8%) as compared to hemorrhagic stroke patients (214, 96.8%) (*p* = 0.09). The proportion of stroke patients with a history of DM, dyslipidemia, obesity, smoking, alcohol consumption, CAD, atrial fibrillation, cardiopathy (CHF), valvular heart disease, previous stroke, and sedentary lifestyle are also shown in Table 1. The prevalence of hyperuricemia among acute stroke patients was 46.6% (327/701). The clinical and laboratory data of patients with ischemic and hemorrhagic stroke were compared (Table 2).

### 3.2. Outcome in the Acute Phase and 90 Days after Stroke

**Figure 1:**
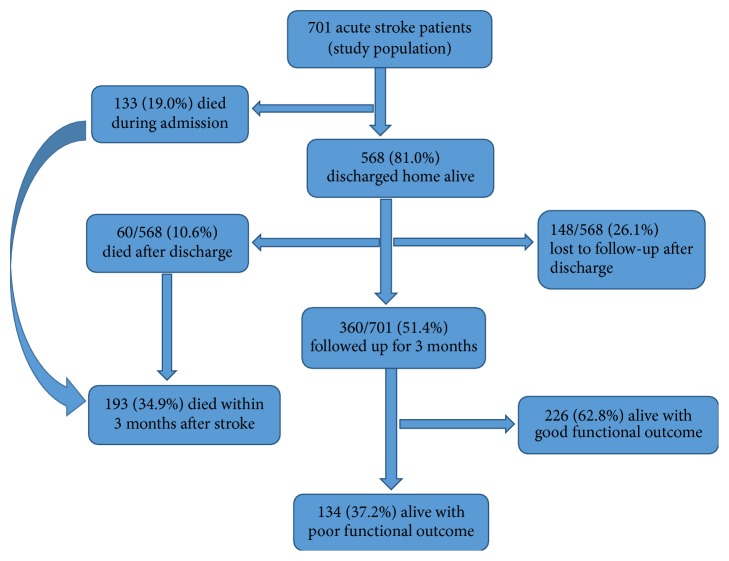
Flow chart of functional outcome and death during the 3-month follow-up.

The outcome of patients within the 90 days after stroke has been assessed in Figure 1. In this series, 133 out of 701 patients (19.0%) died during admission and 568 patients (81.0%) discharged home alive were eligible for follow-up among whom 10.6% (60/568) died within three months and 26.1% (148/568) were lost to follow-up. Among stroke survivors (*n* = 360), 62.8% and 37.2% had good and bad functional outcomes. Overall, the 3-month poststroke mortality was 34.9% (193/701). The mean SUA concentration ± SD (standard deviation) of stroke survivors (65.73 ± 21.96 mg/l) was significantly lower than that of those who died (75.07 ± 28.41 mg/l, *p* value < 0.001). The mean ± SD duration of hospital stay and of follow-up was 8.8 ± 6.2 days and 53.6 ± 40.0 days, respectively. On univariate analysis, there was a significant association between high SUA levels and death among acute stroke patients (*p* < 0.001, OR = 2.067, 95% CI: 1.449–2.950).

### 3.3. Predictive Value of SUA on Mortality among Acute Stroke Patients (Table 3)

**Figure 2:**
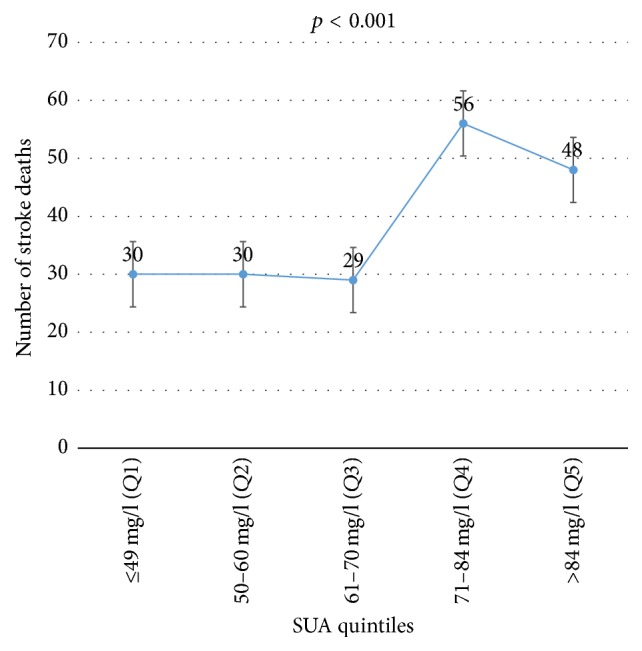
Death rate across SUA quintiles.

**Figure 3:**
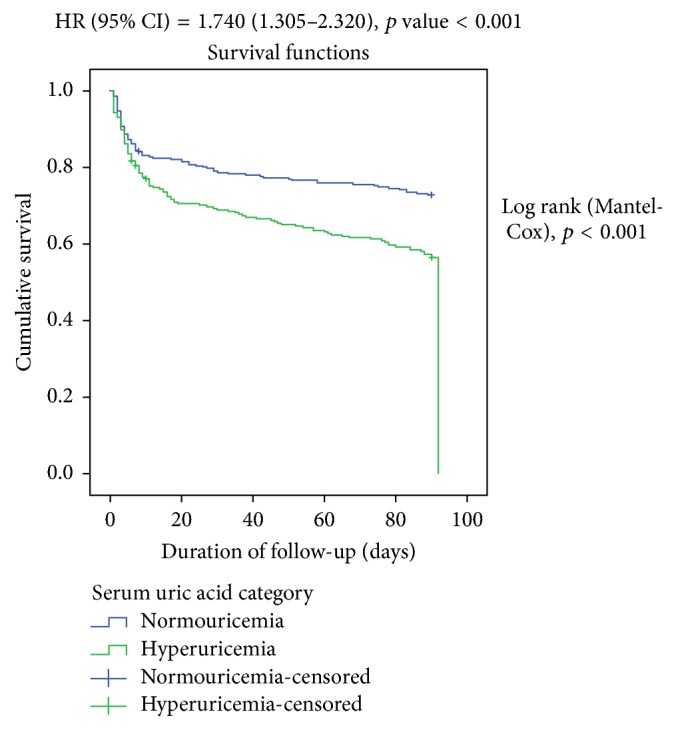
A graph showing a Kaplan-Meier survival rate of patients with normouricemia compared with hyperuricemia after an acute stroke.

On univariate analysis, the following were factors significantly associated with death in acute stroke patients: history of DM, smoking, alcohol abuse, dyslipidemia, atrial fibrillation, cardiomyopathy (CHF), CKD, hemorrhagic stroke type, hyperglycemia, low HDLc, increased triglycerides, high TG, hyperuricemia, GCS less than 9, NIHSS > 14, mRS > 2, and presence of in-hospital complications (*p* < 0.05). In the significant association observed between hyperuricemia and death among acute stroke patients on univariate analysis, patients with elevated SUA after stroke had unadjusted odds ratio (95% CI) of 2.067 (1.449–2.950) (*p* < 0.001) of mortality compared with normouricemic patients. However, on multivariate analysis, no such independent association was seen (OR = 1.265 (CI: 0.707–2.263); *p* = 0.428). After multivariate analysis, only atrial fibrillation, hyperglycemia, hypertriglyceridemia, GCS less than 9, mRS greater than 2, and NIHSS greater than 14 remained as significant independent predictors of death within 3 months after stroke in acute stroke patients (*p* < 0.05). However, an independent association between increasing SUA concentration and survival was noted with the Kaplan-Meier curve with adjusted HR (95% CI) of 1.740 (1.305–2.320), *p* < 0.001, as shown in Figure 2. The proportion of death within 3 months after stroke significantly increased with high levels of SUA (*p* < 0.001) ranging from 30 (15.5%) deaths in the lowest SUA quintile range (*Q*1) to 56 (29.0%) deaths in the 4th quintile range (*Q*4) and 48 deaths (24.5%) in the highest SUA quintile range (*Q*5). The curves in Figure 3 show that death within 3 months after stroke in acute stroke significantly increases as SUA levels increase among stroke patients. This is shown in Figure 3.

### 3.4. Predictive Value of SUA on Functional Outcome among Acute Stroke Patients (Table 4)

On univariate analysis, there was a significant association between high SUA levels and poor outcome among acute stroke patients (*p* < 0.001, OR = 3.268, 95% CI: 2.093–5.104). On univariate analysis, the following were also significant predictors of poor functional outcomes in all acute stroke patients: older patients >45 years, unemployment, recurrent stroke, hyperuricemia, low GCS (≤8), NIHSS > 14, mRS> 2, elevated serum urea, and presence of in-hospital complications (*p* < 0.05). On multivariate analysis, there was a significant association between hyperuricemia and poor functional outcomes among acute stroke patients, where patients with elevated SUA after stroke had adjusted odds ratio of 2.482 (95% CI: 1.399–4.404; *p* = 0.002) of mortality compared with normouricemic patients (Table 4).

## 4. Discussion

In our study, the prevalence of hyperuricemia among acute stroke patients was 46.6% (327/701); we observed that even though high SUA was not an independent predictor of stroke death, death rate significantly increased across the higher SUA quintile ranges in acute stroke patients. The findings from this observational prospective study support the notion that SUA is an adverse prognostic marker of stroke outcome among black African patients.

In Ghana, Sarfo et al. reported a high frequency of hyperuricemia of 46.3% among Ghanaian stroke patients which was similar to that seen in our study [17]. Similarly, Masoud et al., in Iran [18], reported the mean SUA levels in 55 patients (46 ischemic and 9 hemorrhagic stroke patients) studied to be 59.4 ± 17.0 mg/l and about half of the patients (47.3%) were hyperuricemic. In India, Kotwal et al. reported a slightly lower prevalence of hyperuricemia among 100 acute stroke patients in 2015 [19]. This very slight discrepancy could due to the differences in methodology such as timing of SUA measurement. The prevalence of hyperuricemia in the United States' general population is 20.1% according to a large 10-year follow-up study reported by the National Health and Nutrition Examination Survey (NHANES), 2007-2008 [20]. Another study in Bangkok's population showed that prevalence of hyperuricemia is 24.4% [21] and a study in a developing country reported the prevalence of hyperuricemia to be 35.2% in men and 8.7% in women [22]. In India, Koppula et al. showed that 24.7% of controls in their study were hyperuricemic [23]. According to these studies, the prevalence of hyperuricemia is significantly higher in patients with acute stroke than in the normal population.

Regarding the association between stroke mortality and hyperuricemia in our study, we noted that hyperuricemia was associated with stroke mortality and was a predictor of poor functional outcome among acute stroke patients. Similarly, in the recent Ghanaian prospective observational cohort, SUA concentration was positively correlated with stroke severity and an association was observed between stroke mortality and hyperuricemia [17]. In a longitudinal study including 418 patients in Congo, reported by Longo-Mbenza et al., hyperuricemia was found to be a predictor of stroke and all-cause mortality [24]. Karagiannis et al. found that elevated SUA was strongly associated with early death among 435 patients presenting with stroke in Greece [25]. Furthermore, stroke death rate significantly increased across the higher SUA quintile ranges in both ischemic stroke and all stroke patients. Similarly, Strasak et al. in a long-term prospective study demonstrated that the highest SUA quintile concentration was significantly related to mortality from CHF and stroke [26]. Our findings are to a greater extent also consistent with the body of previous scientific evidence which supports the fact that SUA is an adverse predictor of early stroke death and functional outcome [5, 27–30]. In experimental and in vivo conditions, it has been shown that SUA can act as a proinflammatory molecule [31]. A direct mechanistic role for hyperuricemia in atherogenesis and the clinical course of cerebrovascular disease has been suggested by the links between elevated SUA and increased production of oxygen free radicals [32], accelerated oxygenation of low-density lipoproteins which facilitates lipid peroxidation [33, 34], impairment of nitric oxide production with subsequent activation of the renin-angiotensin system [35], and induction of endothelial dysfunction and smooth muscle cell proliferation [36, 37]. Furthermore, uric acid increases platelet adhesiveness and aggregation [32, 38], stimulates the synthesis of monocyte chemoattractant protein-1 (MCP-1) in rat vascular smooth muscles via mitogen-activated protein kinase and cyclooxygenase-2 [39], and stimulates mice mononuclear cells to produce IL-1*β*, IL-6, and tumor necrosis factor-*α* (TNF-*α*) [40]. All these factors are important for the development and progression of atherosclerosis and may therefore explain the strong association between uric acid and carotid atherosclerosis, a well-known risk factor for stroke [8, 41].

Despite the overwhelming evidence from this current and previous epidemiological studies, some studies still demonstrate contradictory findings. In a study carried out by Amaro et al., higher SUA levels were associated with an increased rate of excellent recovery independently of baseline variables [10]. Still in Spain, Chamorro et al. found out that diabetic patients had lower SUA values and there was a 12% increase in the odds of good clinical outcome for each milligram per deciliter increase of SUA in patients with acute ischemic stroke [42]. Wu et al. (2013) conducted a study on 1351 ischemic and 380 cerebral hemorrhage patients, which showed that decreased uric acid levels correlated with poor outcomes in acute ischemic stroke patients, but not in cerebral hemorrhage patients [43]. According to a study conducted in 585 young Chinese patients with acute ischemic stroke by Zhang et al., in 2010, it was observed that elevated SUA is an independent predictor for good clinical outcome of acute cerebral infarction among young adults and, unlike our study, lower SUA levels at time of admission were observed more frequently in the lowest quintile for patients with severe stroke [44]. Therefore, these findings support the fact that high SUA concentration could be neuroprotective and has antioxidant properties in the setting of an acute stroke.

Despite these intriguing differences, the findings of this study support the fact that SUA is a poor prognostic marker or predictor of mortality and functional outcome among acute stroke Cameroonian patients. SUA reduction is a viable and promising strategy to reduce stroke mortality and poor functional outcome.

## 5. Conclusion

The prevalence of hyperuricemia in stroke is quite high as about 1 in 2 acute stroke patients had hyperuricemia on admission. Hyperuricemia is associated with stroke mortality and is a predictor of adverse functional outcomes in acute stroke. These results therefore suggest that prospective studies should be conducted to determine whether reducing SUA levels after acute ischemic stroke would be beneficial within our setting.

## Figures and Tables

**Table 1:** Basic characteristics and cerebrovascular risk factors (CVRFs) of stroke.

Variables	Values
Age, years (mean ± SD)	60.58 ± 13.27
Males, *n* (%)	389 (55.5)
Ischemic stroke, *n* (%)	480 (68.5)
Cardioembolic stroke, *n* (%)	108 (22.5)
Atherosclerosis stroke, *n* (%)	292 (60.8)
Lacunar stroke, *n* (%)	55 (11.5)
Undetermined, *n* (%)	25 (5.2)
Hemorrhagic stroke, *n* (%)	221 (31.5)
Hypertension, *n* (%)	664 (94.7)
Diabetes mellitus, *n* (%)	240 (34.2)
Smoking, *n* (%)	591 (84.3)
Alcohol abuse, *n* (%)	257 (36.7)
Dyslipidemia, *n* (%)	526 (75.0)
Obesity, *n* (%)	203 (29.0)
Metabolic syndrome, *n* (%)	169 (24.1)
Coronary artery disease, *n* (%)	41 (5.8)
Atrial fibrillation, *n* (%)	93 (13.3)
Heart failure (cardiomyopathy), *n* (%)	91 (13.0)
Valvulopathy, *n* (%)	39 (5.6)
Past stroke, *n* (%)	151 (21.5)
Sedentary lifestyle, *n* (%)	130 (18.5)
Family history of stroke, *n* (%)	22 (3.1)
Hyperuricemia, *n* (%)	**327 (46.6)**
Gout, *n* (%)	22 (3.1)
Chronic kidney disease, *n* (%)	43 (6.1)
HIV seropositive, *n* (%)	25 (3.6)

**Table 2:** Baseline clinical profile of acute stroke patients.

Variables	Ischemic stroke	Hemorrhagic stroke	*p* value
Age, mean ± SD	62.77 ± 13.32	55.81 ± 11.86	<0.001
Systolic blood pressure, mean ± SD	159.77 ± 30.532	178.92 ± 34.437	<0.001
Diastolic blood pressure, mean ± SD	95.32 ± 19.389	107.62 ± 22.138	<0.001
Pulse, mean ± SD	83.837 ± 18.7469	86.223 ± 20.3571	0.128
Temperature, mean ± SD	37.329 ± 0.8933	37.772 ± 3.5019	0.010
O_2_ saturation, mean ± SD	95.68 ± 4.764	94.69 ± 5.175	0.027
Height, mean ± SD	4.1672 ± 14.8528	4.0214 ± 13.8822	0.959
Weight, mean ± SD	81.8439 ± 19.052	86.8788 ± 18.989	0.181
Body mass index, mean ± SD	31.8915 ± 6.7657	30.3858 ± 5.6557	0.572
Glasgow Coma Score, mean ± SD	13.42 ± 2.531	11.46 ± 3.866	<0.001
NIHSS Score, mean ± SD	10.42 ± 7.837	16.51 ± 10.177	<0.001
Modified Rankin Score, mean ± SD	2.796 ± 1.5182	3.710 ± 1.3972	<0.001
Serum uric acid, mean ± SD	71.058 ± 25.3186	63.341 ± 22.1664	<0.001
White blood cell count, mean ± SD	7.3827 ± 4.68110	8.5558 ± 4.01643	0.001
Creatinine, mean ± SD	16.1565 ± 26.125	18.0011 ± 43.412	0.487
Urea, mean ± SD	0.5795 ± 1.162	0.6688 ± 1.825	0.435
CRP, mean ± SD	63.155 ± 72.766	74.624 ± 81.649	0.213
ESR, mean ± SD	45.30 ± 40.800	53.61 ± 40.736	0.193
Glycemia, mean ± SD	1.4891 ± 0.89088	1.4185 ± 0.74641	0.307
Glycated hemoglobin, mean ± SD	9.025 ± 10.285	6.802 ± 1.798	0.243
Total cholesterol, mean ± SD	1.9600 ± 0.02665	1.9700 ± 0.2608	0.245
Triglycerides, mean ± SD	1.4054 ± 0.21183	1.1185 ± 0.04916	0.390
HDL cholesterol, mean ± SD	0.4946 ± 0.25125	0.5187 ± 0.21349	0.245
LDL cholesterol, mean ± SD	1.2860 ± 0.51114	1.2941 ± 0.62535	0.864
Hematocrit, mean ± SD	38.478 ± 7.1100	40.460 ± 6.7768	0.001
Length of hospital stay (days)	8.400 ± 6.000	9.720 ± 9.260	0.053
Duration of follow-up (days)	55.330 ± 39.550	49.900 ± 40.861	0.100

**Table 3:** Independent predictors of death within 3 months after stroke.

Predictors	Alive	Died	Unadjusted OR (95% CI)	*p* value	Adjusted OR (95% CI)	*p* value
Age > 45 years	**306 (63.9)**	**173 (18.9)**	**1.527 (0.84**–**2.635)**	**0.127**	0.984 (0.451–2.147)	0.967
Male gender	206 (66.7)	103 (33.3)	0.856 (0.602–1.216)	0.384	—	—
HTN	343 (65.1)	184 (34.9)	1.013 (0.443–2.318)	0.975	—	—
DM	**111 (57.2)**	**83 (42.8)**	**1.693 (1.178**–**2.432)**	**0.004**	0.984 (0.506–1.913)	0.961
Smoking	**70 (76.7)**	**21 (23.1)**	**0.506 (0.300**–**0.853)**	**0.010**	0.839 (0.372–1.895)	0.673
Alcohol	151 (70.9)	62 (29.1)	0.665 (0.454–0.946)	**0.024**	0.768 (0.417–1.412)	0.395
Dyslipidemia	285 (68.8)	129 (31.2)	0.530 (0.358–0.786)	**0.001**	0.602 (0.305–1.187)	0.143
Obesity	113 (71.1)	46 (28.9)	0.684 (0.459–1.019)	**0.061**	0.429 (0.103–1.778)	0.243
METS	**93 (69.9)**	**40 (30.1)**	**0.751 (0.492**–**1.143)**	**0.180**	1.705 (0.359–8.103)	0.502
CAD/IHD	23 (67.6)	11 (32.4)	0885 (0.422–1.858)	0.748	—	—
AFIB	**34 (49.3)**	**35 (50.7)**	**2.124 (1.275**–**3.533)**	**0.003**	**3.198 (1.386**–**7.376)**	**0.006**
Cardiopathy	**36 (52.9)**	**32 (47.1)**	**1.789 (1.072**–**2.986)**	**0.025**	1.129 (0.483–2.642)	0.779
Valvulopathy	20 (66.7)	10 (33.3)	0.929 (0.426–2.027)	0.853	—	—
Previous stroke	71 (62.3)	43 (37.7)	1.167 (0.761–1.788)	0.479	—	—
CKD	**17 (43.7)**	**22 (56.4)**	**2.596 (1.343**–**5.017)**	**0.003**	2.139 (0.829–5.522)	0.116
Hemorrhagic stroke	**104 (53.1)**	**92 (46.9)**	**2.242(1.560**–**3.223)**	**<0.001**	1.211 (0.635–2.307)	0.561
SBP > 140 mmHg	**276 (63.6)**	**158 (36.4)**	**1.574 (0.885**–**2.134)**	**0.156**	1.751 (0.803–3.821)	0.159
DBP > 90 mmHg	**227 (62.4)**	**137 (37.6)**	**1.867 (1.225**–**2.854)**	**0.061**	0.801 (0.405–1.586)	0.525
Hyperglycemia	**107 (52.2)**	**98 (47.8)**	**2.439 (1.699**–**3.502)**	**<0.001**	**2.263 (1.203**–**4.256)**	**0.011**
High LDLc	270 (66.0)	139 (34.0)	0.858 (0.578–1.273)	0.447	—	—
Low HDLc	**210 (70.9)**	**86 (24.7)**	**0.574 (0.403**–**0.817)**	**0.002**	0.673 (0.394–1.148)	0.146
High TC	170 (62.7)	101 (37.3)	1.227 (0.865–1.741)	0.252	—	—
High TG	**70 (51.1)**	**67 (48.9)**	**2.203 (1.484**–**3.270)**	**<0.001**	**1.857 (1.003**–**3.437)**	**0.049**
Hyperuricemia	**148 (56.5)**	**114 (40.7)**	**2.067 (1.449**–**2.950)**	**<0.001**	1.265 (0.707–2.263)	0.428
GCS ≤ 8	**8 (8.7)**	**84 (91.3)**	**33.908(15.916**–**72.242)**	**<0.001**	**7.604 (3.221**–**17.948)**	**<0.001**
NIHSS > 14	**60 (27.6)**	**157 (72.4)**	**21.806(13.820**–**34.407)**	**<0.001**	**6.733 (3.659**–**12.390)**	**<0.001**
mRS > 2	**184 (50.0)**	**184 (50.0)**	**19.556(9.708**–**39.392)**	**<0.001**	**3.363 (1.456**–**7.767)**	**0.005**
Complications	**108 (46.6)**	**124 (53.4)**	**4.193 (2.894**–**6.075)**	**<0.001**	1.631 (0.943–2.821)	0.080

METS: metabolic syndrome; AFIB: atrial fibrillation; CKD: chronic kidney disease; CAD/IHD: coronary artery/ischemic heart disease.

**Table 4:** Independent predictors of poor functional outcome within 3 months post stroke.

Predictors	Good outcome	Poor outcome	Unadjusted OR (95% CI)	*p* value	Adjusted OR (95% CI)	*p* value
Age > 45 years	185 (60.5)	13 (24.1)	2.063 (1.061–4.010)	**0.030**	2.306 (0.966–5.508)	0.060
Male gender	132 (64.1)	74 (35.4)	0.878 (0.571–1.352)	0.555	—	—
Unemployed	123 (68.0)	58(32.0)	0.639 (0.416–0.983)	**0.041**	1.075 (0.593–1.948)	0.811
Unmarried (single)	62(68.1)	29 (31.9)	0.731(0.441–1.210)	0.222	—	—
No health insurance	67 (67.7)	32 (32.3)	0.745 (0.457–1.214)	0.236	—	—
HTN	212 (61.8)	131(38.2)	2.884 (0.813–10.226)	0.087	2.860 (0.670–12.211)	0.156
DM	66 (59.5)	45 (40.5)	1.226 (0.775–1.940)	0.385	—	—
Smoking	40 (57.1)	30 (42.9)	1.341 (0.789–2.281)	0.277	—	—
Alcohol	95 (62.9)	56 (37.1)	0.990 (0.642–1.527)	0.964	—	—
Dyslipidemia	175 (61.4)	110 (38.6)	1.336 (0.778–2.294)	0.293	—	—
Obesity	69 (61.1)	44(38.9)	1.112 (0.703–1.759)	0.649	—	—
METS	56 (60.2)	37 (39.8)	1.158 (0.713–1.880)	0.553	—	—
CAD/IHD	16 (69.6)	7 (30.4)	0.723 (0.290–1.806)	0.486	—	—
AFIB	20 (58.8)	14 (41.2)	1.202 (0.585–2.467)	0.616	—	—
Cardiopathy	25 (69.4)	11 (30.6)	0.719 (0.342–1.512)	0.383	—	—
Valvulopathy	14(70.0)	6 (30.0)	0.710 (0.266–1.894)	0.492	—	—
Recurrent stroke	32 (45.1)	39 (54.9)	2.488 (1.468–4.220)	**0.001**	**2.566 (1.247**–**5.280)**	**0.010**
CKD	11 (64.7)	6 (35.3)	0.916 (0.331–2.537)	0.866	—	—
Hemorrhagic stroke	63 (60.6)	41 (39.4)	1.141 (0.714–1,822)	0.582	—	—
SBP > 140 mmHg	174 (63.0)	102 (37.0)	0.953 (0.576–1.576)	0.850	—	—
DBP > 90 mmHg	142 (62.6)	85 (37.4)	1.026 (0.659–1.598)	0.909	—	—
Hyperglycemia	59 (55.1)	48 (44.9)	1.580 (0.996–2.506)	0.051	1.253 (0.670–0.670)	0.480
High LDLc	165 (61.1)	105 (38.9)	1.339 (0.808–2.219)	0.257	—	—
Low HDLc	129 (61.4)	81 (38.6)	1.149 (0.744–1.776)	0.531	—	—
High TC	106 (62.4)	64 (37.6)	1.035 (0.675–1.588)	0.875	—	—
High TG	45 (64.3)	25 (35.7)	0.823 (0.536–1.589)	0.771	—	—
Hyperuricemia	69 (46.6)	79 (53.4)	3.268 (2.093–5.104)	**<0.001**	**2.482 (1.399**–**4.404)**	**0.002**
GCS < 9	1 (12.5)	7 (87.5)	12.402 (1.509–101.943)	**0.005**	4.306 (0.439–42.263)	0.210
NIHSS > 14	18 (30.0)	42 (70.0)	5.275 (2.883–9.653)	**<0.001**	1.369 (0.657–2.854)	0.402
mRS > 2	67 (36.4)	117 (63.6)	16.333 (9.114–29.269)	**<0.001**	**14.639 (7.591**–**28.232)**	**<0.001**
Complications	53 (49.1)	55 (50.9)	2.273 (1.432–3.606)	**<0.001**	1.033 (0.552–1.936)	0.919
High urea	40 (50.0)	40 (50.0)	1.968 (1,189–3.257)	**0.008**	1.037 (0.532–2.020)	0.916
High creatinine	56 (58.9)	39 (41.1)	1.239 (0.767–2.002)	0.381	—	—

METS: metabolic syndrome; AFIB: atrial fibrillation; CKD: chronic kidney disease; CAD/IHD: coronary artery/ischemic heart disease.
